# Identification of microbial agents in tissue specimens of ocular and periocular sarcoidosis using a metagenomics approach

**DOI:** 10.12688/f1000research.55090.1

**Published:** 2021-08-17

**Authors:** Amde Selassie Shifera, Christopher Pockrandt, Natalia Rincon, Yuchen Ge, Jennifer Lu, Ales Varabyou, Anne E. Jedlicka, Karen Sun, Alan L. Scott, Charles Eberhart, Jennifer E. Thorne, Steven L. Salzberg

**Affiliations:** 1 Wilmer Eye Institute, Johns Hopkins University, Baltimore, MD, USA; 2 Center for Computational Biology, Johns Hopkins University, Baltimore, MD, USA; 3 Department of Biomedical Engineering, Johns Hopkins University, Baltimore, MD, USA; 4 Department of Computer Science, Johns Hopkins University, Baltimore, MD, USA; 5 Genomic Analysis and Sequencing Core Facility, Johns Hopkins Bloomberg School of Public Health, Baltimore, MD, USA; 6 Department of Microbiology & Immunology, Johns Hopkins Bloomberg School of Public Health, Baltimore, MD, USA; 7 Department of Epidemiology, Johns Hopkins Bloomberg School of Public Health, Baltimore, MD, USA

**Keywords:** sarcoidosis, ocular sarcoidosis, orbital sarcoidosis, metagenomics, next-generation sequencing, pathogen discovery, Campylobacter concisus, Neisseria elongate, Streptococcus salivarius, Pseudopropionibacterium propionicum, Paracoccus yee; Exophiala oligosperma, Lomentospora prolificans, Aspergillus versicolor, Mupapillomavirus 1

## Abstract

**Background**: Metagenomic sequencing has the potential to identify a wide range of pathogens in human tissue samples. Sarcoidosis is a complex disorder whose etiology remains unknown and for which a variety of infectious causes have been hypothesized. We sought to conduct metagenomic sequencing on cases of ocular and periocular sarcoidosis, none of them with previously identified infectious causes.

**Methods**: Archival tissue specimens of 16 subjects with biopsies of ocular and periocular tissues that were positive for non-caseating granulomas were used as cases. Four archival tissue specimens that did not demonstrate non-caseating granulomas were also included as controls. Genomic DNA was extracted from tissue sections. DNA libraries were generated from the extracted genomic DNA and the libraries underwent next-generation sequencing.

**Results**: We generated between 4.8 and 20.7 million reads for each of the 16 cases plus four control samples. For eight of the cases, we identified microbial pathogens that were present well above the background, with one potential pathogen identified for seven of the cases and two possible pathogens for one of the cases. Five of the eight cases were associated with bacteria ( *Campylobacter concisus, Neisseria elongata, Streptococcus salivarius, Pseudopropionibacterium propionicum, *and *Paracoccus yeei*), two cases with fungi ( *Exophiala oligosperma, Lomentospora prolificans and Aspergillus versicolor*) and one case with a virus (Mupapillomavirus 1). Interestingly, four of the five bacterial species are also part of the human oral microbiome.

**Conclusions**: Using a metagenomic sequencing we identified possible infectious causes in half of the ocular and periocular sarcoidosis cases analyzed. Our findings support the proposition that sarcoidosis could be an etiologically heterogenous disease. Because these are previously banked samples, direct follow-up in the respective patients is impossible, but these results suggest that sequencing may be a valuable tool in better understanding the etiopathogenesis of sarcoidosis and in diagnosing and treating this disease.

## Introduction

Sarcoidosis is a systemic inflammatory disease characterized by the formation of non-caseating granulomas in the affected tissues ^1^. Although sarcoidosis can affect almost any organ in the human body, it most commonly affects the lungs, the skin and the ocular and periocular tissues. The frequency of ocular and periocular involvement in patients with sarcoidosis ranges between 25% and 60% depending on the particular population studied ^2^. The manifestations of ocular and periocular sarcoidosis include uveitis, conjunctival granulomas, eyelid granulomas, orbital inflammation, dacryoadenitis, dacryocystitis, scleritis and optic neuropathy. Ocular and periocular sarcoidosis accounts for about 5% of patients seen in a uveitis practice and it results in blindness in at least one eye in approximately 10% of the affected patients ^2^.

In spite of numerous investigations that have been carried out since the first case of sarcoidosis was reported in 1877 by Jonathan Hutchinson ^3^, the etiology of sarcoidosis remains unknown. However, there is very strong evidence that supports the assertion that the pathogenesis of sarcoid granulomas involves an oligoclonal CD4 T cell-mediated immune response to a persistent antigen, most likely an exogenous antigen derived from microbial or inanimate sources ^4– 6^. Microbial sources of antigens that have been suspected of causing sarcoidosis include bacteria such as mycobacteria, *Propionibacterium acnes*, *Tropheryma whipplei* and *Borrelia burgdorferi,* fungi such as *Coccidioides spp.*, and viruses such as Epstein-Barr virus, cytomegalovirus and hepatitis C virus ^4– 10^. It is possible that different antigens could be involved in different patients, resulting in a diverse pattern of organ involvement, natural history and clinical course. In addition to exposure to the requisite antigen, it is believed that the disease occurs only within the appropriate genetic background of the host ^5^.

The availability of next-generation sequencing (NGS) technologies has opened vast opportunities for pathogen discovery in human disease ^11^. We hypothesized that metagenomic sequencing using NGS would identify pathogen-derived microbial DNA within sarcoid granulomas. We conducted a metagenomics analysis on DNA extracted from archival tissue specimens of 16 cases of ocular and periocular sarcoidosis, and we detected possible microbial pathogens in eight of the cases. We anticipate that the identification of potential microbial etiologies of sarcoidosis may lead to large-scale metagenomics studies that can be validated by pathogen isolation followed by investigations to establish the pathogenic role of the suspected microorganisms in the causation of sarcoidosis.

## Methods

### Ethics statement

The Johns Hopkins University School of Medicine Institutional Review Board (IRB) approved this study (approval number IRB00126932), which was undertaken in accordance with the principles of the Declaration of Helsinki and in compliance with the Health Insurance Portability and Accountability Act. The study was categorized by the IRB as ‘Not Human Subjects Research’ and as such obtaining informed consent was not required.

### Collection of demographic, clinical and histopathological data

Demographic, clinical and histopathological data (from initial presentation to subsequent follow-ups) were retrospectively collected for each study subject by reviewing the electronic medical records of the subjects. For each subject, the data (including sex, age, race, diagnosis, and results of microbiological, histopathological and radiologic tests) were gathered in a de-identified manner before analysis was carried out.

### Human tissue specimens

Paraffin-embedded archival tissue specimens of subjects who had biopsies of ocular and periocular tissues at the Wilmer Eye Institute of Johns Hopkins Hospital during the period between February 2010 and February 2017, and that were positive for non-caseating granulomas, were included in the study. A total of 18 such specimens were identified: six specimens of orbital tissues, two specimens of eyelid tissues, two specimens of the lacrimal sac, five specimens of the conjunctiva, one specimen of the cornea and two specimens of the globe. In addition, a total of four archival tissue specimens (one from the conjunctiva and three from the lacrimal gland) that did not demonstrate non-caseating granulomas were included to be used as controls.

For each archival tissue specimen, 10 sections, each 10 µm thick, were cut from the paraffin blocks and used for DNA extraction. Two of the conjunctival specimens that were positive for non-caseating granulomas were excluded from the study due to poor quality of the DNA extracted from the specimens. Therefore, a total 16 positive specimens and four negative specimens were used.

### DNA extraction

Genomic DNA was extracted from paraffin-embedded tissue sections using the QIAamp DNA FFPE tissue kit and deparaffinization solution according to the manufacturer’s (Catalog numbers 56404 and 19093, respectively, Qiagen, Valencia, CA, USA) recommended and supplementary protocols. Quality of DNA was assessed by Genomic ScreenTape analysis on a TapeStation 2200 (Agilent Technologies, Santa Clara, CA, USA). The Quant-iT PicoGreen dsDNA reagent kit (Catalog number P7589, Invitrogen/ThermoFisher Scientific, Waltham, MA, USA) was used for quantitation of DNA samples, with fluorescent reads performed on a SpectraMax M2 plate reader (Molecular Devices, San Jose, CA, USA).

### Generation of DNA library

Libraries were prepared from ten nanograms of DNA using the Ovation Ultralow V2 DNA-seq Library Preparation kit (Catalog number 0344, Tecan Genomics, Redwood City, CA, USA). The recommended protocol was followed with the exception of the initial fragmentation step. Fragmentation was performed enzymatically, instead of ultrasonically, using Celero fragmentation buffer and Celero fragmentation enzyme from the Celero PCR Workflow with Enzymatic Fragmentation kit (Catalog number 9363, Tecan Genomics). Fragmentation time was optimized to 10 minutes, and a modified purification was performed with AMPure XP beads (Catalog number A63881, Beckman Coulter, Brea, CA, USA). Library amplification was performed for 13 cycles based on the Manufacturer’s recommendation for starting input amount of DNA (10 ng), in an Applied Biosystems GeneAmp 9700 or Veriti thermal cycler (ThermoFisher Scientific). Cycling parameters were: 72°C for two minutes, 95°C for three minutes, (98°C 20 sec, 65°C 30 sec, 72°C 30 sec) for 13 cycles, 72°C for one minute, and a 4°C hold. Amplification primers and enzyme were part of the Ovation Ultralow V2 kit. Quality of purified libraries was assessed by D1000 ScreenTape analysis on a TapeStation 2200, with region analysis performed for sizing. Quantitation of libraries was performed by qPCR with the Kapa Library Quantitation kit for Illumina (Catalog number KK4824/07960140001, Roche, Basel, Switzerland) in an Applied Biosystems StepOne Plus Real Time PCR system (ThermoFisher Scientific). A six-point standard curve, with a concentration range of 20 pM to 0.0002 pM was run, as per the Kapa recommended protocol. Run parameters were an initial denaturation at 95°C for 5 minutes and 35 cycles (95°C 30 sec denaturation and 60°C 45 sec annealing/extension/data acquisition), followed by a ramp from 65°C to 95°C for melt curve analysis. qPCR results and sizing data were imported to the Kapa Library Quantitation Data Template for calculations of library concentrations and yields. Libraries were diluted to 10 nM, and an equimolar pool prepared. A final quality assessment of the library pool was performed by High Sensitivity DNA Lab Chip Analysis on a BioAnalyzer 2100 (Agilent Technologies), and a final quantity check was performed on a Qubit Flex Fluorometer using Qubit High Sensitivity DNA reagents and standards (Catalog number Q32854, Invitrogen/ThermoFisher Scientific).

### Next-generation sequencing

Sequencing of the library pool was performed with a 300 cycle (2x150 bp) SP run on an Illumina NovaSeq6000 sequencer (Illumina, San Diego, CA, USA) at Johns Hopkins Genomics, Genetic Resources Core Facility, RRID:SCR_018669.

### Analysis of metagenomics data

For each of the 20 metagenomics samples, we first removed all human sequences by aligning all paired reads to the GRCh38 human reference genome using Bowtie2 ^12^ in very-sensitive mode. To ensure removal of all human sequences, we removed an entire read pair if either of the read mates aligned to the human reference.

For each patient, we generated two runs of 150-bp paired-end sequencing data. For simplicity, we concatenated the reads by merging the two runs from each patient. We then compared all patient samples against a KrakenUniq ^13^ database consisting of 5,981 bacterial species (18,484 genomes), 295 archaeal species (374 genomes), 9,905 viral species (10,012 genomes), 250 eukaryotic pathogen (e.g. fungi, amoebas) species (388 genomes), the human GRCh38.p13 genome, and vector sequences. The total numbers of reads per sample, along with the numbers identified as microbial, are shown in Table 1.

**Table 1.** Number of reads sequenced for each of the samples in this study. Microbial reads include all reads identified as bacteria, fungi, other eukaryotic pathogens, or viruses. Samples 119, 120, 122, and 123 are controls.

Sample	Total number of reads	Microbial reads
101	11,776,007	521,477
102	9,550,287	871,188
103	8,995,205	113,599
104	19,120,004	313,655
105	7,802,252	973,926
106	11,561,273	179,288
107	11,408,189	493,991
108	10,957,245	177,035
109	4,765,078	49,002
112	5,752,886	242,711
113	20,719,130	2,782,033
114	15,580,774	621,465
115	11,252,011	1,800,606
116	14,273,179	2,539,876
117	12,300,035	472,053
118	9,066,395	402,393
119	8,779,112	256,073
120	6,253,953	233,615
122	6,410,090	66,718
123	5,613,806	35,532

KrakenUniq ^13^ classifies each read by breaking reads into overlapping k-mers, searching the database for the lowest common ancestor of each k-mer, and then assigning the overall read a taxon based on the k-mer taxon distribution. Unlike Kraken 1 ^14^ and Kraken 2 ^15^, KrakenUniq reports for every taxonomic classification - not only the read counts but also the number of distinct k-mers, giving extra confidence in classification. Hits with a low count of distinct k-mers are often false positives; e.g., due to low-complexity repetitive sequences in the genome of a pathogen.

In order to detect outlier read counts among the metagenomics samples, we used a modified Z-score calculation as defined by Iglewicz and Hoaglin ^16^. As compared to a normal Z-score calculation which uses mean values that may be influenced by extreme outliers, this formula uses the median deviation and the sample median. The formula for the modified Z-score for sample *i* is as follows:

Modified Z-score_i = 0.6745*(X_i - X_median) / MAD

where X_median is the median read count across all samples and MAD is the median absolute deviation. The median absolute deviation (MAD) is defined as the median of the absolute difference of the observation from the sample median:

MAD = median(|X_i - X_median|)

Reads from species with a significant modified Z-score and a high distinct k-mer count were then extracted and aligned to the NCBI nucleotide database to verify whether they were true positives or whether they hit other species equally well or better, suggesting a false positive match.

### Analysis of candidate pathogen reads found in control samples

For 7/9 candidate infectious microbes, we found small numbers of reads, ranging from 1–64, in one or more control samples. For 8/9 of these pathogens, we found small numbers of reads in other non-control samples. In order to clarify why these reads were present, we analyzed them to determine if they were either (a) computational false positives or (b) possible cross-contamination in the multiplexed sequencing experiment. In addition to counting reads, KrakenUniq counts the number of unique k-mers (k=31 in our experiments) found in each species in a sample ^13^. Each 150-bp read may contain as many as 130 unique 31-mers, if the hit is a true positive and if each k-mer is distinct. For all of the candidate infectious agents, the number of unique k-mers per read was quite high, ranging from 50 to >100. If the unique kmer count for a read is low, the read may consist of low-complexity, repetitive sequence, suggesting that the match is a computational false positive. To check for this possibility, from each of the control samples that had reads matching a candidate infectious agent, we aligned those reads using BLAST ^17^ against NCBI’s comprehensive “nr” nucleotide database. If the reads hit the genome of the candidate pathogen, that suggested cross-contamination in the sample. If the reads matched other genomes or did not match the genome of interest, that suggested they were false positives.

This evaluation found that small levels of cross-contamination explained the control sample matches for seven of the eight candidate pathogens identified in Table 3, as follows. (1) Kraken identified 0-4 reads as *Campylobacter concisus* in the control samples, and BLAST alignments confirmed that they matched *C. concisus*, suggesting a small amount of cross-contamination. (2) For *Neisseria elongata*, Kraken found 1-14 reads in the control samples, and all were confirmed by BLAST *.* (3) For *Exophiala oligosperma*, we found 1-2 reads in the controls and all were confirmed by BLAST *.* (4) For *Streptococcus salivarius*, we found 3-33 reads in the control samples, and we confirmed a random sample of them using BLAST *.* (5) We found 2-13 reads matching *Pseudopropionibacterium propionicum* in the control samples, and all were confirmed by BLAST. (6) We found 1-8 reads in the control samples matching *Aspergillus versicolor* and confirmed a random sample of them by BLAST. (7) We found 2-64 reads matching *Paracoccus yeei* in the control samples and all were confirmed by BLAST *.* (8) For *Lomentospora prolificans*, we found 0 reads in the control samples; however, Kraken identified 1-66 reads in the non-control samples. We searched a sample of these reads against “nr” using BLAST, and all aligned to different species while none had BLAST alignments to *L. prolificans.* Upon further inspection, all the reads had a very low number of unique k-mers. Thus, we determined that these reads were low complexity, repetitive sequences that yielded false positive matches.

## Results

### Demographic and clinical data

The demographic and clinical data of the patients (16 cases and 4 controls) whose archival tissue specimens were used in the study are presented in Table 2. The cases ranged in age from 32 to 79 years while the controls ranged in age from 38 to 71 years. Among the cases, 13 were female and three were male, while among the controls three were female and one male. Seven of the cases were diagnosed to have systemic sarcoidosis while none of the controls were reported to have systemic sarcoidosis.

**Table 2.** Demographic and clinical data of the cases and controls *.

Sample	*Age*	*Sex*	*Race*	*Nature of* *specimen*	*Affected tissue*	*Histopathologic* *findings*	*Presence* *of systemic* *sarcoidosis*
**101**	72	F	Black	Excisional biopsy	Orbital tissue	Non-caseating granulomas	Pulmonary sarcoidosis
**102**	56	M	White	Excisional biopsy	Extraocular muscle	Non-caseating granulomas	None reported
**103**	32	F	Black	Excisional biopsy	Orbital tissue	Non-caseating granulomas	Pulmonary sarcoidosis
**104**	75	F	Black	Excisional biopsy	Lacrimal sac	Non-caseating granulomas	Pulmonary & cutaneous sarcoidosis
**105**	50	F	Black	Excisional biopsy	Eyelid	Non-caseating granulomas	Pulmonary & cardiac sarcoidosis
**106**	74	F	White	Excisional biopsy	Orbital tissue	Non-caseating granulomas	None reported
**107**	65	F	White	Excisional biopsy	Orbital tissue	Non-caseating granulomas	None reported
**108**	51	F	Black	Excisional biopsy	Lacrimal sac	Non-caseating granulomas	None reported
**109**	50	F	Black	Excisional biopsy	Orbital tissue	Non-caseating granulomas	None reported
**112**	72	F	Black	Excisional biopsy	Conjunctiva	Non-caseating granulomas	None reported
**113**	79	M	White	Excisional biopsy	Conjunctiva	Non-caseating granulomas	None reported
**114**	58	F	Black	Excisional biopsy	Conjunctiva	Non-caseating granulomas	None reported
**115**	33	F	Black	Excisional biopsy	Cornea #	Non-caseating granulomas	Pulmonary sarcoidosis
**116**	49	F	White	Excisional biopsy	Eyelid	Non-caseating granulomas	Neuro- sarcoidosis
**117**	38	M	Black	Enucleated globe	Iris; ciliary body; retina; choroid	Non-caseating granulomas	Cutaneous sarcoidosis
**118**	38	F	Black	Enucleated globe	Choroid	Non-caseating granulomas	None reported
***119***	*38*	*M*	*Black*	*Excisional* *biopsy*	*Conjunctiva*	*Conjunctival* *inclusion cyst with* *adjacent lacrimal* *tissue*	*None reported*
***120***	*54*	*F*	*Black*	*Excisional* *biopsy*	*Lacrimal gland*	*Chronic* *non-specific* *dacryoadenitis*	*None* *reported*
***122***	*68*	*F*	*White &* *Hispanic*	*Excisional* *biopsy*	*Lacrimal gland*	*Chronic* *non-specific* *dacryoadenitis*	*None* *reported*
***123***	*71*	*F*	*Asian*	*Excisional* *biopsy*	*Lacrimal gland*	*IgG4* *dacryoadenitis*	*None* *reported*

*The rows with roman text represent the cases whereas the rows with italicized text represent the controls. #This patient also had sarcoidosis-associated panuveitis of the ipsilateral eye.

**Table 3.** Number of reads identified in each sample for species identified as possible pathogens. For each column, the value in bold text is significantly higher than any other value in that column.

Sample	*Campylobacter concisus*	*Mupapillomavirus 1*	*Neisseria elongata*	*Exophiala oligosperma*	*Streptococcus salivarius*	*Pseudopropionibacterium* *propionicum*	*Lomentospora prolificans*	*Aspergillus versicolor*	*Paracoccus yeei*
**101**	**179**	0	34	65	50	29	9	5	31
**102**	0	**49**	12	2	10	5	0	16	44
**103**	0	0	13	3	9	21	0	1	7
**104**	0	0	8	4	3	12	0	0	30
**105**	4	0	33	13	13	7	66	7	35
**106**	2	0	6	7	5	1	0	1	28
**107**	1	0	**675**	1	7	19	1	14	29
**108**	0	0	4	2	2	6	0	7	8
**109**	0	0	34	2	9	6	2	0	5
**112**	2	0	14	**11965**	16	15	0	0	40
**113**	7	0	207	14	**1829**	60	4	2	204
**114**	5	0	15	2	4	**4840**	1	0	48
**115**	4	0	95	4	26	29	**367**	**585**	98
**116**	6	0	43	10	18	66	0	19	134
**117**	3	0	23	2	13	11	0	1	**7780**
**118**	2	0	14	3	20	5	3	2	43
**119**	1	0	2	2	17	5	0	4	25
**120**	4	0	14	1	13	4	0	1	64
**122**	0	0	1	1	0	2	0	8	39
**123**	1	0	1	1	3	13	0	0	2

### Metagenomics analysis

We identified pathogens that were possibly associated with disease in eight of the 16 case samples ( Table 3). For seven of the samples, a possible pathogen species was present at a much higher level than in any of the controls or the other clinical samples, and for one sample (sample 115), two possible pathogens were identified. For each of the eight samples and nine pathogens, the read counts for the pathogen were statistically higher than expected based on the distribution of read counts in all other samples. We measured this expectation using a modified z-score, which represents the number of standard deviations above the mean for the read count from the possible pathogen (see Methods). Below we briefly discuss each of the eight samples in which possible infectious agents were detected.

**Sample 101**. Sample 101 contained 179 read pairs from *Campylobacter concisus*, while no other sample had more than seven read pairs, which could be cross-contamination from the multiplexed sequencing run. The controls had 0-4 reads ( Table 2). This is a highly significant finding, with a modified z-score of 119.

**Sample 102:** Sample 102 was notable for the presence of 49 read pairs from Mupapillomavirus 1, more commonly known as human papillomavirus type 1 (HPV 1). Strikingly, none of the other 19 samples had even a single read from this virus. We confirmed that all of the reads represented HPV 1, and that they covered ~3000 bp of this small (7811 bp) genome. Thus, the virus was clearly present in this sample, and this sample only.

**Sample 107:** Sample 107 contained 675 reads from *Neisseria elongata*. Most other case samples had very few reads from this bacterium, although sample 113 had 207 reads. The control samples had just 1-14 reads, suggesting that sample 107 had a clear excess from this species (modified z-score 37.2).

**Sample 112:** Sample 112 was noteworthy for having a strikingly large burden of sequence from the fungus *Exophiala oligosperma*, a known although somewhat unusual human pathogen ^18^. *E. oligosperma* had a far higher count in sample 112 than in any other sample, with 11,965 read pairs, compared to just 0 to 14 reads in other samples, with the exception of sample 101 that had 65 reads. Alignment of the reads to the genome indicates that they cover approximately one million base pairs from the 38 megabase genome of this fungus, and thus they are (as expected) randomly dispersed throughout the genome.

**Sample 113:** Sample 113 contained 1,829 reads from *Streptococcus salivarius*, far more than were found in any other samples (modified z-score 175). Read counts in other samples ranged from 2 to 50, and the controls had 2 to 13.

**Sample 114:** Sample 114 contained 4,840 reads from *Pseudopropionibacterium propionicum*, a pathogen that is sometimes dismissed because it is mistaken for *Propionibacterium acnes*, a common skin bacterium ^19^. Until 2016, the two bacterium were both placed in the genus Propionibacterium, at which point *P. propionicum* was re-classified into a distinct genus. Despite the similar name, *P. propionicum* causes very different types of infections. All other samples had fewer than 20 reads from this species, yielding a modified z-score of 465.

**Sample 115:** Sample 115 had 367 read pairs with near-perfect matches to the pathogenic fungus *Lomentospora prolificans*. Fewer than 10 reads from this fungus were found in other samples, except for sample 105 which had 66 reads. The small number of reads in other samples here (and in other cases) might represent cross-contamination between samples.

**Sample 115:** Sample 115 was the only sample with two candidate pathogens, both fungi. In addition to *L. prolificans*, sample 115 had 585 reads from *Aspergillus versicolor*. These reads are unambiguous matches to the genome, and all other samples had 20 or fewer matches to this fungus.

**Sample 117:** Sample 117 had 7,780 reads from *Paracoccus yeei*, a bacterial pathogen. Although *P. yeei* was detected in other samples, no other sample had more than 204 reads. Those might represent cross-contamination in the multiplexed sequencing run, given the far higher read count (modified z-score 402) in sample 117. Alignment to the genome demonstrated that the reads were well dispersed, covering 340 Kb of the 4.7 Mbp genome.

### Histopathological data

**Figure 1.**
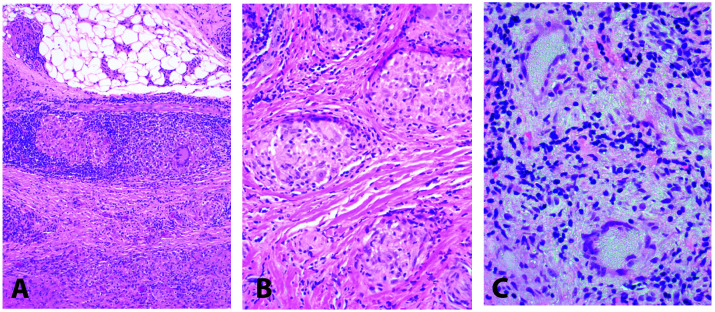
Histopathology with hematoxylin and eosin staining. Light microscopy revealed non-caseating granulomatous inflammation in the orbit ( **A, B**) and conjunctiva ( **C**). Original magnifications 100x ( **A**), 200x ( **B**), 400x ( **C**). A was from sample 107; B was from sample 101; C was from sample 114. These images were selected for illustrative purposes and the images were all obtained by the diagnostic pathology laboratory at the Johns Hopkins Hospital as part of routine medical care. For histopathological examination, briefly, paraffin sections 5 µm thick were cut and stained with hematoxylin and eosin using standard protocols by the pathology laboratory.

Histopathological examination carried out as part of routine medical care of all the cases showed typical non-caseating granulomas. Representative histopathological images from three of the eight cases that were positive for microbial DNA are presented in Figure 1. Except for specimen 115, the seven other cases were negative on acid-fast and fungal stains at the time of initial histopathological evaluation. Specimen 115 did not undergo staining for acid-fast and fungi at the time of initial histopathological examination of the specimen (which was the same specimen used in our study) that was obtained from the patient during a corneal transplant procedure. However, this case underwent another corneal transplant procedure eight months after the initial transplant and the specimen obtained at the time, while still showing non-caseating granulomas, was negative on acid-fast and fungal stains.

## Discussion

In this study we conducted a metagenomics analysis of DNA extracted from archival tissue specimens that were obtained from 16 cases with ocular or periocular sarcoidosis and identified DNA evidence of a possible microbial pathogen in eight of the cases. The microbial agents identified from the tissue specimens were five species of bacteria ( *Campylobacter concisus, Neisseria elongata, Streptococcus salivarius, Pseudopropionibacterium propionicum,* and *Paracoccus yeei*), three species of fungi ( *Exophiala oligosperma, Lomentospora prolificans and Aspergillus versicolor*) and one species of virus (Mupapillomavirus 1).

The case that was positive for *Campylobacter concisus* DNA had orbital and pulmonary sarcoidosis. *C. concisus* is a Gram-negative bacterium that colonizes the oral cavity of humans ^20, 21^. Currently, humans are the only known hosts of this bacterium ^20, 21^. A few studies have found an association between *C. concisus* and Barrett’s esophagus ^22, 23^. In addition, recent studies have also demonstrated association between Crohn’s disease and *C. concisus*, which could translocate from the oral cavity to the intestine ^24, 25^. It is plausible that *C. concisus* could be aspirated from the oral cavity to the lungs, and then also potentially to distant organs such as the orbit, where it could incite an inflammatory process.

The case that was positive for *Neisseria elongata* DNA had orbital sarcoidosis with no systemic sarcoidosis reported. *N. elongata* is a Gram-negative bacterium that is part of the normal flora of the oral cavity ^26^. There are a number of case reports of infective endocarditis associated with colonization by *N. elongata* ^26– 29^. In addition, the bacterium has been implicated in some cases of osteomyelitis ^29, 30^.

The case in which *Streptococcus salivarius* DNA was detected had conjunctival sarcoidosis without reported evidence of systemic sarcoidosis. *S. salivarius* is a Gram-positive bacterium which is part of the normal flora of the oral cavity ^31^. It establishes itself in the human oral cavity within a few hours after birth and persists as a predominant inhabitant of the oral cavity ^32^. The bacterium has been associated with invasive infections including meningitis ^31^, bacteremia ^33^ and prosthetic joint infection ^34^. Interestingly, *S. salivarius* has also been associated with exogenous endophthalmitis following keratoplasty with a contaminated donor cornea ^35^ and after an intravitreal injection ^36^.

*Pseudopropionibacterium propionicum* (formerly known as *Propionibacterium propionicum*, *Arachnia propionica* and *Actinomyces propionicus*) DNA was detected in a case that had conjunctival sarcoidosis without reported systemic sarcoidosis. *P. propionicum* is a Gram-positive bacterium that is part of the human oral flora ^37^. It has been associated with human infectious diseases that resemble actinomycosis. There are case reports of the bacterium being associated with lacrimal canaliculitis, cervicofacial infections ^38, 39^, tympanomastoiditis ^40^, pulmonary and thoracic infections ^19, 41, 42^, osteomyelitis ^43^ and brain abscess ^44^. Infection by *P. propionicum* causes chronic granulomatous inflammation characterized by abscesses, draining sinuses and fibrosis ^19, 45^.

*Paracoccus yeei* DNA was detected in a patient who had sarcoidosis that involved the iris, ciliary body, choroid and retina; this case did have a reported evidence of cutaneous sarcoidosis. *P. yeei* is a Gram-negative bacterium that is found naturally in soil and brine ^46^. In a study involving 1321 patients with idiopathic uveitis, Drancourt *et al.* detected *P. yeei* in one patient by conducting 16S rDNA sequencing on an intraocular fluid specimen ^47^. In another, study *P. yeei* was cultured from the aqueous humor of a patient who had developed corneal graft rejection ^48^. In addition, *P. yeei* has been associated with peritonitis in a patient undergoing peritoneal dialysis ^49^ and with cutaneous infection followed by bacteremia in a patient with heart failure ^50^.

The case in which *Exophiala oligosperma* DNA was detected had conjunctival sarcoidosis with no reported systemic sarcoidosis. *E. oligosperma* is a dimorphic fungus that has been associated with cutaneous and subcutaneous lesions ^18^ and olecranon bursitis ^51^. *Exophilia* species have been isolated from the skin, cutaneous tissues, the heart, the lungs, bone and the central nervous system ^51– 54^. Interestingly, a member of the genus *Exophiala* ( *E. jeanselmei*) has been associated with keratitis ^55^ and another member ( *E. dermatitidis*) with endophthalmitis ^56^.

The case in which DNA belonging to each of *Lomentospora prolificans* and *Aspergillus versicolor* was simultaneously detected had corneal sarcoidosis with reported pulmonary sarcoidosis; in addition, the case had sarcoidosis-associated panuveitis of the affected eye. *L. prolificans* is an anamorphic fungus that has been associated with localized bone and joint infections in the immunocompetent host and with disseminated disease (involving the lungs, the ears, the eyes and the central nervous system) in the immunocompromised host ^57, 58^. *A. versicolor* is a filamentous fungus. It has been associated with invasive pulmonary aspergillosis ^59^, onychomycosis ^60^ and endogenous endophthalmitis ^61^.

The case that was positive for Mupapillomavirus 1 had orbital sarcoidosis that involved the extraocular muscle tissues with no systemic sarcoidosis reported. Mupapillomavirus 1 is a double-stranded DNA virus that belongs to the virus family Papillomaviridae. It has been isolated from plantar warts ^62^ and from punctate keratotic lesions of the foot ^63^. Interestingly, the virus has also been detected, using a PCR method, in the lesions of cutaneous sarcoidosis in a patient who also had pulmonary sarcoidosis ^64^. In addition, other human papillomaviruses have been associated with ocular diseases, including pterygium and ocular surface squamous neoplasia ^65^.

In this study, we have identified nine different microorganisms in eight cases of ocular and periocular sarcoidosis. It is not known at this time if any of these microorganisms play any role in the causation of sarcoidosis. The microbial agents could gain access to the ocular and periocular tissues directly from the environment (especially after trauma or surgery) or could reach this tissues via hematogenous spread after initial colonization of distant tissues such as the lungs, the skin and the subcutaneous tissues. It is interesting to note that four of the five bacterial species that were identified by our study are also part of the human oral microbiome. In those cases, the oral cavity could be the source of the microorganisms that involved the ocular and periocular tissues.

One perplexing finding of our study is that none of the nine microorganisms were detected in more than one case. A possible explanation for this observation is that sarcoidosis is an etiologically heterogenous disease. In support of this argument, it is important to note that sarcoidosis, in addition to being associated with a number of microbial agents, has also been linked to a number of inanimate sources of antigens, including tattoo ink, aluminum, zirconium, talc, and insecticides ^5, 6^.

Another limitation of our study is that potential RNA viruses could not be detected due to the nature of the assay. The relatively small sample size and the fact that paraffin-embedded archival tissue specimens were used are also additional shortcomings. Future studies using a metagenomics approach on a much larger sample size and employing fresh tissue specimens from a variety of sources are recommended.

## Conclusions

In this study, using a metagenomics approach, we identified nine potential microbial agents in tissue specimens of eight cases of ocular and periocular sarcoidosis. The role of these microorganisms in the causation of sarcoidosis is not clear at this time. Our study has limitations due to the relatively small sample size and due to the fact that metagenomics analysis was carried out on archival tissue specimens. Large-scale metagenomics studies using fresh tissue specimens are needed to provide a better understanding of the potential role of microbial agents in the causation of sarcoidosis. The results of such studies could lead to improved means for the diagnosis and treatment of sarcoidosis.

## Data availability

### Underlying data

NCBI BioProject: Metagenomics sequencing of infectious microbes from ocular sarcoidosis tissue specimens. Accession number PRJNA745199; https://identifiers.org/NCBI/bioproject:PRJNA745199.

## References

[1] Chen ES Moller DR : Sarcoidosis--scientific progress and clinical challenges. *Nat Rev Rheumatol.* 2011;7(8):457–67. 10.1038/nrrheum.2011.93 21750528

[2] Groen F Rothova A : Ocular involvement in sarcoidosis. *Br J Ophthalmol.* 2000;84(1):110–6. 10.1055/s-0037-1602382 10611110 PMC1723211

[3] James DG : Centenary commemoration of sarcoidosis and of Jonathan Hutchinson. *Br Med J.* 1969;2(5649):109–10. 10.1136/bmj.2.5649.109 4887040 PMC1982866

[4] Saidha S Sotirchos ES Eckstein C : Etiology of sarcoidosis: does infection play a role? *Yale J Biol Med.* 2012;85(1):133–41. 22461752 PMC3313528

[5] Chen ES Moller DR : Etiology of sarcoidosis. *Clin Chest Med.* 2008;29(3):365–77, vii. 10.1016/j.ccm.2008.03.011 18539232

[6] Gerke AK Hunninghake G : The immunology of sarcoidosis. *Clin Chest Med.* 2008;29(3):379–90, vii. 10.1016/j.ccm.2008.03.014 18539233

[7] Newman KL Newman LS : Occupational causes of sarcoidosis. *Curr Opin Allergy Clin Immunol.* 2012;12(2):145–50. 10.1097/ACI.0b013e3283515173 22314258 PMC4196683

[8] Newman LS Rose CS Bresnitz EA : A case control etiologic study of sarcoidosis: environmental and occupational risk factors. *Am J Respir Crit Care Med.* 2004;170(12):1324–30. 10.1164/rccm.200402-249OC 15347561

[9] Kuberski T Yourison I : Coccidioidomycosis A Cause of Sarcoidosis. *Open Forum Infect Dis.* 2017;4(3):ofw117. 10.1093/ofid/ofw117 28717672 PMC5502954

[10] Nikoskelainen J Hannuksela M Palva T : Antibodies to Epstein-Barr virus and some other herpesviruses in patients with sarcoidosis, pulmonary tuberculosis and erythema nodosum. *Scand J Infect Dis.* 1974;6(3):209–16. 10.3109/inf.1974.6.issue-3.01 4370734

[11] Calistri A Palu G : Editorial commentary: Unbiased next-generation sequencing and new pathogen discovery: undeniable advantages and still-existing drawbacks. *Clin Infect Dis.* 2015;60(6):889–91. 10.1093/cid/ciu913 25572900

[12] Langmead B Salzberg SL : Fast gapped-read alignment with Bowtie 2. *Nat Methods.* 2012;9(4):357–9. 10.1038/nmeth.1923 22388286 PMC3322381

[13] Breitwieser FP Baker DN Salzberg SL : KrakenUniq: confident and fast metagenomics classification using unique k-mer counts. *Genome Biol.* 2018;19(1):198. 10.1186/s13059-018-1568-0 30445993 PMC6238331

[14] Wood DE Salzberg SL : Kraken: ultrafast metagenomic sequence classification using exact alignments. *Genome Biol.* 2014;15(3):R46. 10.1186/gb-2014-15-3-r46 24580807 PMC4053813

[15] Wood DE Lu J Langmead B : Improved metagenomic analysis with Kraken 2. *Genome Biol.* 2019;20(1):257. 10.1186/s13059-019-1891-0 31779668 PMC6883579

[16] Iglewicz B Hoaglin D : Volume 16: How to Detect and Handle Outliers.In: Mykytka EF, editor. The ASQC Basic References in Quality Control: Statistical Techniques: ASQC Quality Press; 1993. Reference Source

[17] Altschul SF Madden TL Schaffer AA : Gapped BLAST and PSI-BLAST: a new generation of protein database search programs. *Nucleic Acids Res.* 1997;25(17):3389–402. 10.1093/nar/25.17.3389 9254694 PMC146917

[18] Rimawi BH Rimawi RH Mirdamadi M : A case of Exophiala oligosperma successfully treated with voriconazole. *Med Mycol Case Rep.* 2013;2:144–7. 10.1016/j.mmcr.2013.08.003 24432241 PMC3885957

[19] Suzuki H Arshava EV Ford B : Don't Let Its Name Fool You: Relapsing Thoracic Actinomycosis Caused by Pseudopropionibacterium propionicum (Formerly Propionibacterium propionicum). *Am J Case Rep.* 2019;20:1961–5. 10.12659/AJCR.919775 31884507 PMC6956836

[20] Mahendran V Tan YS Riordan SM : The prevalence and polymorphisms of zonula occluden toxin gene in multiple Campylobacter concisus strains isolated from saliva of patients with inflammatory bowel disease and controls. *PLoS One.* 2013;8(9):e75525. 10.1371/journal.pone.0075525 24086553 PMC3781098

[21] Liu F Ma R Wang Y : The Clinical Importance of *Campylobacter concisus* and Other Human Hosted *Campylobacter* Species. *Front Cell Infect Microbiol.* 2018;8:243. 10.3389/fcimb.2018.00243 30087857 PMC6066527

[22] Blackett KL Siddhi SS Cleary S : Oesophageal bacterial biofilm changes in gastro-oesophageal reflux disease, Barrett's and oesophageal carcinoma: association or causality? *Aliment Pharmacol Ther.* 2013;37(11):1084–92. 10.1111/apt.12317 23600758

[23] Macfarlane S Furrie E Macfarlane GT : Microbial colonization of the upper gastrointestinal tract in patients with Barrett's esophagus. *Clin Infect Dis.* 2007;45(1):29–38. 10.1086/518578 17554697

[24] Zhang L Budiman V Day AS : Isolation and detection of *Campylobacter concisus* from saliva of healthy individuals and patients with inflammatory bowel disease. *J Clin Microbiol.* 2010;48(8):2965–7. 10.1128/JCM.02391-09 20519479 PMC2916630

[25] Chung HK Tay A Octavia S : Genome analysis of *Campylobacter concisus* strains from patients with inflammatory bowel disease and gastroenteritis provides new insights into pathogenicity. *Sci Rep.* 2016;6:38442. 10.1038/srep38442 27910936 PMC5133609

[26] Haddow LJ Mulgrew C Ansari A : *Neisseria elongata* endocarditis: case report and literature review. *Clin Microbiol Infect.* 2003;9(5):426–30. 10.1046/j.1469-0691.2003.00533.x 12848758

[27] Humbert MV Christodoulides M : Atypical, Yet Not Infrequent, Infections with *Neisseria* Species. *Pathogens.* 2019;9(1):10. 10.3390/pathogens9010010 31861867 PMC7168603

[28] Youssef D Marroush TS Levine MT : Endocarditis due to *Neisseria elongata*: A case report and review of the literature. *Germs.* 2019;9(4):188–92. 10.18683/germs.2019.1176 32042725 PMC6942657

[29] Wong JD Janda JM : Association of an important Neisseria species, Neisseria elongata subsp. nitroreducens, with bacteremia, endocarditis, and osteomyelitis. *J Clin Microbiol.* 1992;30(3):719–20. 10.1128/jcm.30.3.719-720.1992 1551990 PMC265139

[30] Spielman AF Ghumman A Panthaki Z : *Neisseria elongata* osteomyelitis: Literature review and case report in a 63-year-old male presenting with progressive right-handed redness, swelling and pain. *Int J Surg Case Rep.* 2020;73:228–30. 10.1016/j.ijscr.2020.07.022 32717677 PMC7385039

[31] Wilson M Martin R Walk ST : Clinical and laboratory features of *Streptococcus salivarius* meningitis: a case report and literature review. *Clin Med Res.* 2012;10(1):15–25. 10.3121/cmr.2011.1001 21817122 PMC3280456

[32] Kaci G Goudercourt D Dennin V : Anti-inflammatory properties of *Streptococcus salivarius*, a commensal bacterium of the oral cavity and digestive tract. *Appl Environ Microbiol.* 2014;80(3):928–34. 10.1128/AEM.03133-13 24271166 PMC3911234

[33] Gautam M Chopra KB Douglas DD : *Streptococcus salivarius* bacteremia and spontaneous bacterial peritonitis in liver transplantation candidates. *Liver Transpl.* 2007;13(11):1582–8. 10.1002/lt.21277 17969206

[34] Olson LB Turner DJ Cox GM : *Streptococcus salivarius* Prosthetic Joint Infection following Dental Cleaning despite Antibiotic Prophylaxis. *Case Rep Infect Dis.* 2019;2019:8109280. 10.1155/2019/8109280 31143483 PMC6501194

[35] Heidemann DG Dunn SP Haimann M : Streptococcus *salivarius* endophthalmitis from contaminated donor cornea after keratoplasty. *Am J Ophthalmol.* 1989;107(4):429–30. 10.1016/0002-9394(89)90672-7 2648851

[36] Chen E Lin MY Cox J : Endophthalmitis after intravitreal injection: the importance of viridans streptococci. *Retina.* 2011;31(8):1525–33. 10.1097/IAE.0b013e318221594a 21878800

[37] Bowden GHW : *Actinomyces, Propionibacterium propionicus,* and *Streptomyces* . In: th Baron S, editors. Medical Microbiology. Galveston (TX)1996. 21413327

[38] Pulverer G Schutt-Gerowitt H Schaal KP : Human cervicofacial actinomycoses: microbiological data for 1997 cases. *Clin Infect Dis.* 2003;37(4):490–7. 10.1086/376621 12905132

[39] Novak A Brutsch P : Case report of actinomycosis caused by Arachnia propionica. *Infection.* 1980;8 Suppl 2:S209–11. 10.1007/BF01639900 7450871

[40] Miglets AW Branson D : Arachnia propionica (Actinomyces propionicus) as an unusual agent in tympanomastoiditis. *Arch Otolaryngol.* 1983;109(6):410–2. 10.1001/archotol.1983.00800200056015 6847502

[41] Brock DW Georg LK Brown JM : Actinomycosis caused by Arachnia propionica: report of 11 cases. *Am J Clin Pathol.* 1973;59(1):66–77. 10.1093/ajcp/59.1.66 4120179

[42] Karnik AM Elhag KM Fenech FF : Arachnia propionica pneumonia in hairy cell leukaemia. *Br J Dis Chest.* 1988;82(4):418–20. 10.1016/0007-0971(88)90098-8 3256353

[43] Conrad SE Breivis J Fried MA : Vertebral osteomyelitis, caused by Arachnia propionica and resembling actinomycosis. Report of a case. *J Bone Joint Surg Am.* 1978;60(4):549–53. 670281

[44] Chau AM Xu LL Fairhall JM : Brain abscess due to Propionibacterium propionicum in Eisenmenger syndrome. *Med J Aust.* 2012;196(8):525–6. 10.5694/mja11.10768 22571312

[45] Smego RA Jr Foglia G : Actinomycosis. *Clin Infect Dis.* 1998;26(6):1255–61; quiz 62-3. 10.1086/516337 9636842

[46] Daneshvar MI Hollis DG Weyant RS : *Paracoccus yeeii* sp. nov. (formerly CDC group EO-2), a novel bacterial species associated with human infection. *J Clin Microbiol.* 2003;41(3):1289–94. 10.1128/JCM.41.3.1289-1294.2003 12624070 PMC150304

[47] Drancourt M Berger P Terrada C : High prevalence of fastidious bacteria in 1520 cases of uveitis of unknown etiology. *Medicine (Baltimore).* 2008;87(3):167–76. 10.1097/MD.0b013e31817b0747 18520326

[48] Kanis MJ Oosterheert JJ Lin S : Corneal graft rejection complicated by *Paracoccus yeei* infection in a patient who had undergone a penetrating keratoplasty. *J Clin Microbiol.* 2010;48(1):323–5. 10.1128/JCM.01798-09 19889897 PMC2812284

[49] Arias MA Clark J : *Paracoccus yeei* as a cause of peritoneal dialysis peritonitis in the United Kingdom. *IDCases.* 2019;15:e00486. 10.1016/j.idcr.2019.e00486 30701158 PMC6348235

[50] Funke G Frodl R Sommer H : First comprehensively documented case of *Paracoccus yeei* infection in a human. *J Clin Microbiol.* 2004;42(7):3366–8. 10.1128/JCM.42.7.3366-3368.2004 15243119 PMC446255

[51] Bossler AD Richter SS Chavez AJ : *Exophiala oligosperma* causing olecranon bursitis. *J Clin Microbiol.* 2003;41(10):4779–82. 10.1128/JCM.41.10.4779-4782.2003 14532219 PMC254319

[52] Kenney RT Kwon-Chung KJ Waytes AT : Successful treatment of systemic *Exophiala dermatitidis* infection in a patient with chronic granulomatous disease. *Clin Infect Dis.* 1992;14(1):235–42. 10.1093/clinids/14.1.235 1571438

[53] Sudduth EJ Crumbley 3rd AJ Farrar WE : Phaeohyphomycosis due to Exophiala species: clinical spectrum of disease in humans. *Clin Infect Dis.* 1992;15(4):639–44. 10.1093/clind/15.4.639 1420677

[54] Uijthof JM de Hoog GS de Cock AW : Pathogenicity of strains of the black yeast *Exophiala (Wangiella) dermatitidis*: an evaluation based on polymerase chain reaction. *Mycoses.* 1994;37(7–8):235–42. 10.1111/j.1439-0507.1994.tb00419.x 7739652

[55] Saeedi OJ Iyer SA Mohiuddin AZ : *Exophiala jeanselmei* keratitis: case report and review of literature. *Eye Contact Lens.* 2013;39(6):410–2. 10.1097/ICL.0b013e3182993901 24045832

[56] Homa M Manikandan P Saravanan V : *Exophiala dermatitidis* Endophthalmitis: Case Report and Literature Review. *Mycopathologia.* 2018;183(3):603–9. 10.1007/s11046-017-0235-4 29374798

[57] Hospenthal DR : 270@ Uncommon Fungi and Related Species. Mandell, Douglas, and Bennett's Principles and Practice of Infectious Diseases. 2015;3003–15.

[58] Kelly M Stevens R Konecny P : Lomentospora prolificans endocarditis--case report and literature review. *BMC Infect Dis.* 2016;16:36. 10.1186/s12879-016-1372-y 26822980 PMC4731902

[59] Charles MP Noyal MJ Easow JM : Invasive pulmonary aspergillosis caused by Aspergillus versicolor in a patient on mechanical ventilation. *Australas Med J.* 2011;4(11):632–4. 10.4066/AMJ.2011.905 23386878 PMC3562921

[60] Torres-Rodriguez JM Madrenys-Brunet N Siddat M : Aspergillus versicolor as cause of onychomycosis: report of 12 cases and susceptibility testing to antifungal drugs. *J Eur Acad Dermatol Venereol.* 1998;11(1):25–31. 9731962

[61] Perri P Campa C Incorvaia C : Endogenous *Aspergillus versicolor* endophthalmitis in an immuno-competent HIV-positive patient. *Mycopathologia.* 2005;160(3):259–61. 10.1007/s11046-005-6871-0 16205976

[62] Danos O Katinka M Yaniv M : Molecular cloning, refined physical map and heterogeneity of methylation sites of papilloma virus type 1a DNA. *Eur J Biochem.* 1980;109(2):457–61. 10.1111/j.1432-1033.1980.tb04815.x 6250842

[63] Egawa K Delius H Matsukura T : Two novel types of human papillomavirus, HPV 63 and HPV 65: comparisons of their clinical and histological features and DNA sequences to other HPV types. *Virology.* 1993;194(2):789–99. 10.1006/viro.1993.1320 8389082

[64] Noparstak M McDaniel B King J : Verrucous sarcoidosis associated with human papillomavirus infection: A case report. *JAAD Case Rep.* 2015;1(5):247–50. 10.1016/j.jdcr.2015.05.006 27051743 PMC4809230

[65] Di Girolamo N : Association of human papilloma virus with pterygia and ocular-surface squamous neoplasia. *Eye (Lond).* 2012;26(2):202–11. 10.1038/eye.2011.312 22134594 PMC3272209

