# Supplementary material for: Identification of microbial agents in tissue specimens of ocular and periocular sarcoidosis using a metagenomics approach
Source: F1000Res. 2021 Aug 17;10:820. [Version 1] doi: 10.12688/f1000research.55090.1 (PMC9515606; doi:10.12688/f1000research.55090.1)
Supplement: Peer review history

# Peer review: Reviewer response for version 1

**DOI:** 10.5256/f1000research.58632.r141758

**Published:** 2022-09-27

**Authors:** Eric B. Suhler

**Affiliations:** 1 Department of Ophthalmology, Oregon Health and Science University, Portland, OR, USA

This article is scientifically sound and proposes a provocative and potentially highly fruitful area of future research into the etiopathogenesis and potential novel treatment of sarcoidosis. Sarcoidosis, while often appropriately presented as an etiologic diagnosis in and of itself, is in fact an idiopathic diagnosis with typical etiologic and histopathologic findings related to presumed exposure to an unknown inciting and possibly infectious antigen. The authors followed this current understanding and applied modern microbiologic techniques to attempt to ascertain more specifically what etiologic agents might be the culprit inciting organisms and found numerous candidates, suggesting that phenotypic sarcoidosis may be triggered by multiple inciting organisms, and laying the groundwork for future prospective studies on specific causative agents, which in the future might lay the groundwork for novel and more effective therapeutics.

Is the work clearly and accurately presented and does it cite the current literature?

Yes

If applicable, is the statistical analysis and its interpretation appropriate?

Yes

Are all the source data underlying the results available to ensure full reproducibility?

Yes

Is the study design appropriate and is the work technically sound?

Yes

Are the conclusions drawn adequately supported by the results?

Yes

Are sufficient details of methods and analysis provided to allow replication by others?

Yes

Reviewer Expertise:

Uveitis, clinical ocular immunology, clinical trials

I confirm that I have read this submission and believe that I have an appropriate level of expertise to confirm that it is of an acceptable scientific standard.

# Peer review: Reviewer response for version 1

**DOI:** 10.5256/f1000research.58632.r138258

**Published:** 2022-06-06

**Authors:** Lynn M. Hassman

**Affiliations:** 1 Department of Ophthalmology and Visual Sciences, Washington University School of Medicine, St. Louis, MO, USA

Shifera *et al*. present a well written analysis of microbial DNA in 16 cases previously diagnosed as ocular or periocular sarcoidosis based on the gold standard histopathologic demonstration of non-caseating granulomas without demonstrable pathogens. They apply next-generation sequencing (NGS )and metagenomic analysis to 16 archival specimens from cases diagnosed as sarcoidosis along with 4 control samples. Based on significant enrichment in specific microbial sequences, they identified 8/16 cases and 0/4 controls with a likely microbial association, including one case in which 2 organisms were detected. Notably, none of the cases were positive for the same microbial sequence enrichment. Each case is presented in the manuscript with a succinct and informative description of the associated microorganism.

 In light of the advancement in pathogen detection afforded by NGS, this study calls into question the diagnosis of sarcoidosis in some of their cases, a diagnosis which requires the absence of detectable pathogens. This is particularly salient in the presented case with corneal sarcoidosis which required 2 corneal transplants, in which DNA from 2 fungal organisms were detected. This case reads suspiciously like infectious keratitis, a more common diagnosis than sarcoid keratitis.

 On the other hand, the presence of microbial DNA is not absolute evidence for a causal infection and caution must be applied in presuming an infectious etiology. Consistent with this, a prevailing theory in the etiology of sarcoidosis is that it represents an aberrant immunologic reaction to antigenic remnants of a prior infection, rather than to an active, ongoing infection.

 In the future, NGS, combined with positive response to appropriate therapy, may improve diagnosis of infectious causes of ocular and periocular inflammation, potentially shrinking the number of cases deemed “sarcoidosis”.

 This study is well-written and informative and its data demonstrate the utility of NGS for identifying either potential microbial triggers of sarcoidosis or potential infectious causes of granulomatous ocular and periocular inflammation. The only criticism is the discussion reads as a suggestion that infection may be causal in sarcoidosis. If this is the author’s position, it should be cited with supporting literature. Otherwise, minor revision to discuss the uncertainty that above cases might represent actual infection vs an immunologic response to a prior infection (i.e. sarcoidosis) is warranted.

Is the work clearly and accurately presented and does it cite the current literature?

Yes

If applicable, is the statistical analysis and its interpretation appropriate?

Yes

Are all the source data underlying the results available to ensure full reproducibility?

Yes

Is the study design appropriate and is the work technically sound?

Yes

Are the conclusions drawn adequately supported by the results?

Yes

Are sufficient details of methods and analysis provided to allow replication by others?

Yes

Reviewer Expertise:

ocular immunology

I confirm that I have read this submission and believe that I have an appropriate level of expertise to confirm that it is of an acceptable scientific standard.
